# Supplementary material for: Astroglial Cell-to-Cell Interaction with Autoreactive Immune Cells in Experimental Autoimmune Encephalomyelitis Involves P2X7 Receptor, β3-Integrin, and Connexin-43
Source: Cells. 2023 Jul 5;12(13):1786. doi: 10.3390/cells12131786 (PMC10340259; doi:10.3390/cells12131786)
Supplement: Supplementary file 1 [file cells-12-01786-s001.zip › cells-2370189-supplementary.pdf]

Supplementary Information

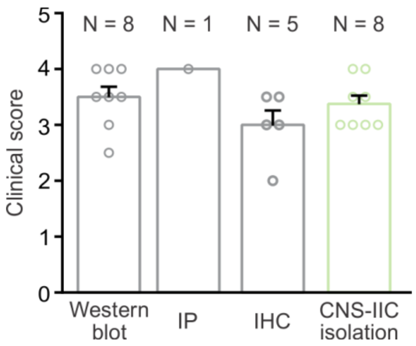

Supplementary Figure S1. Clinical scores of EAE animals used in experiments. Graph shows average clinical scores of EAE animals used in Western blot, immunoprecipitation (IP), immunohistochemistry (IHC), and CNS-IIC isolation experiments. CNS-IIC isolation bar comprises Ca^{2+} imaging and ATP assay experiments. Data are presented as mean ± SEM. Dots represent individual animals. N is the number of animals.

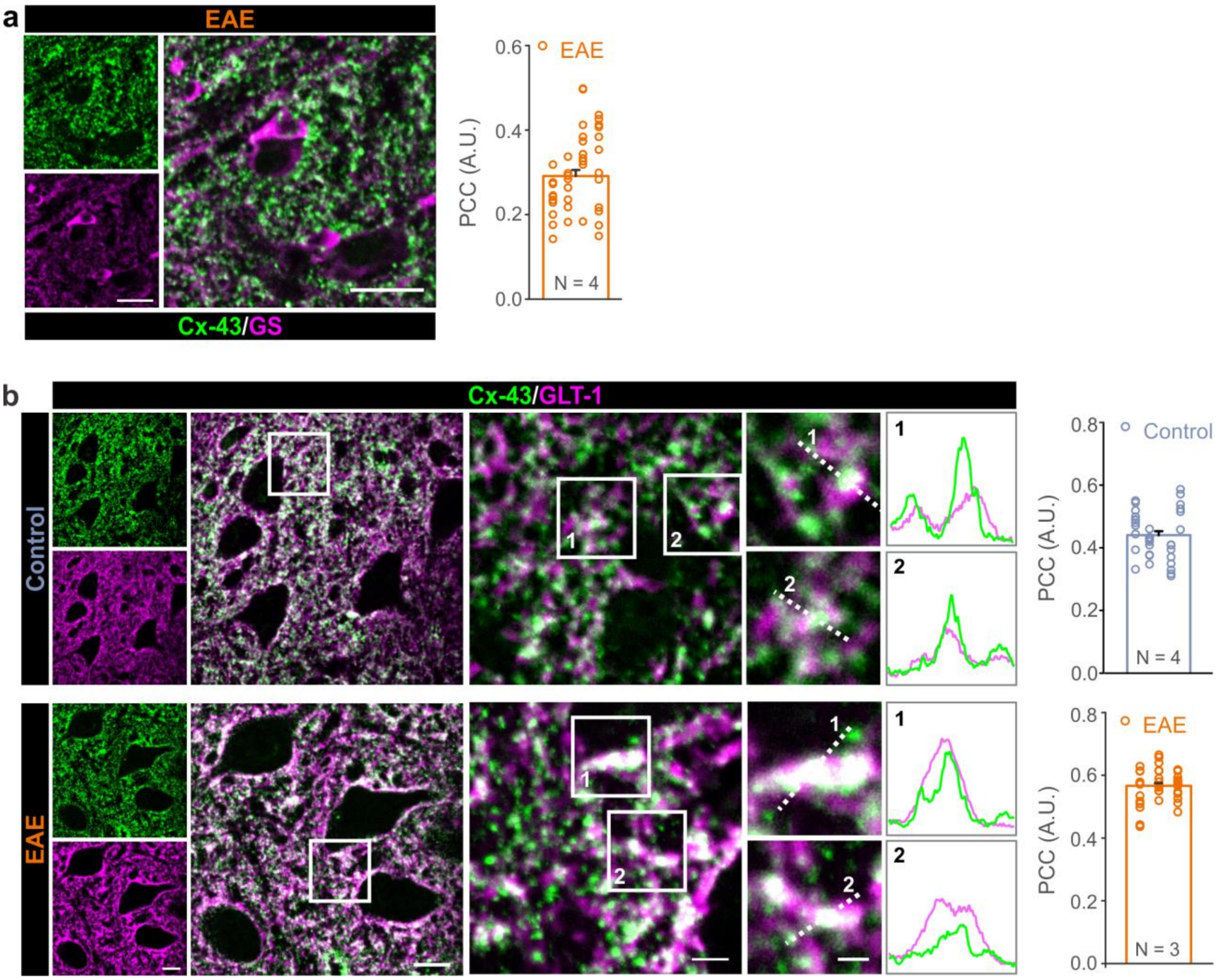

Supplementary Figure S2. Connexin-43 colocalizes with astrocytic markers glutamine synthetase and glutamate transporter-1. (a) Left: Confocal images of the lumbar spinal cord grey matter immunostained for connexin-43 (Cx-43, green) and glutamine synthetase (GS, magenta) in EAE. Right: Graph showing Pearson's correlation coefficient (PCC) of colocalization between Cx-43 and GS. **(b)** Confocal images of the lumbar spinal cord grey matter immunostained for Cx-43 (green) and glutamate transporter-1 (GLT-1, magenta) in Control and EAE. White rectangle corresponds to the enlarged region shown on the right, scale bar 5 µm. Numbered (1, 2) rectangles correspond to the regions presented on the right side, scale bar 2 µm. Profile intensity plots of Cx-43 and GLT-1 fluorescent signals are measured along each white dotted line. Graphs show PCC of colocalization between Cx-43 and GLT-1 in Control and EAE. N indicates number of analyzed animals. Each vertical dot plot corresponds to the data points obtained from the individual animal. Data are presented as mean ± SEM.

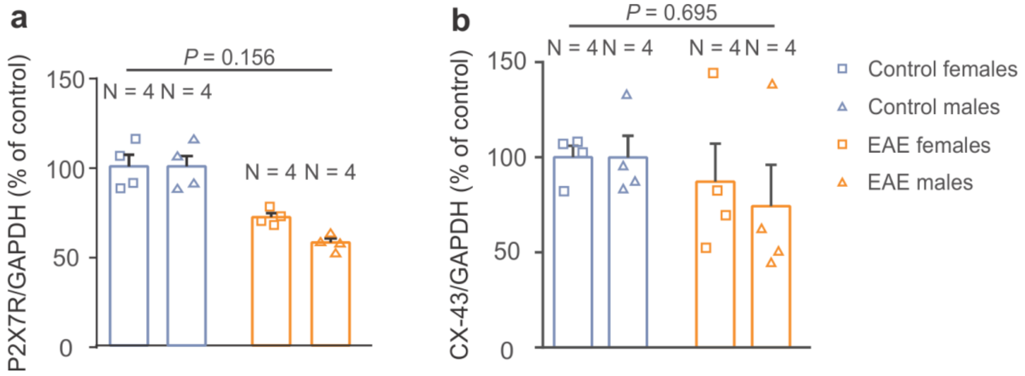

Supplementary Figure S3. P2X7R and connexin-43 expression in EAE females and males. (**a, b**) Graphs showing comparisons of P2X7R (**in a**) and connexin-43 (Cx-43, **in b**) expression in the lumbar spinal cord of female and male rats in control and at the peak of EAE (two-way ANOVA, P = 0.156 for P2X7R and P = 0.695 for Cx-43). Data are shown as mean ± SEM. Triangles represent data obtained for individual females, squares for individual males. Number of animals is displayed on the graph.

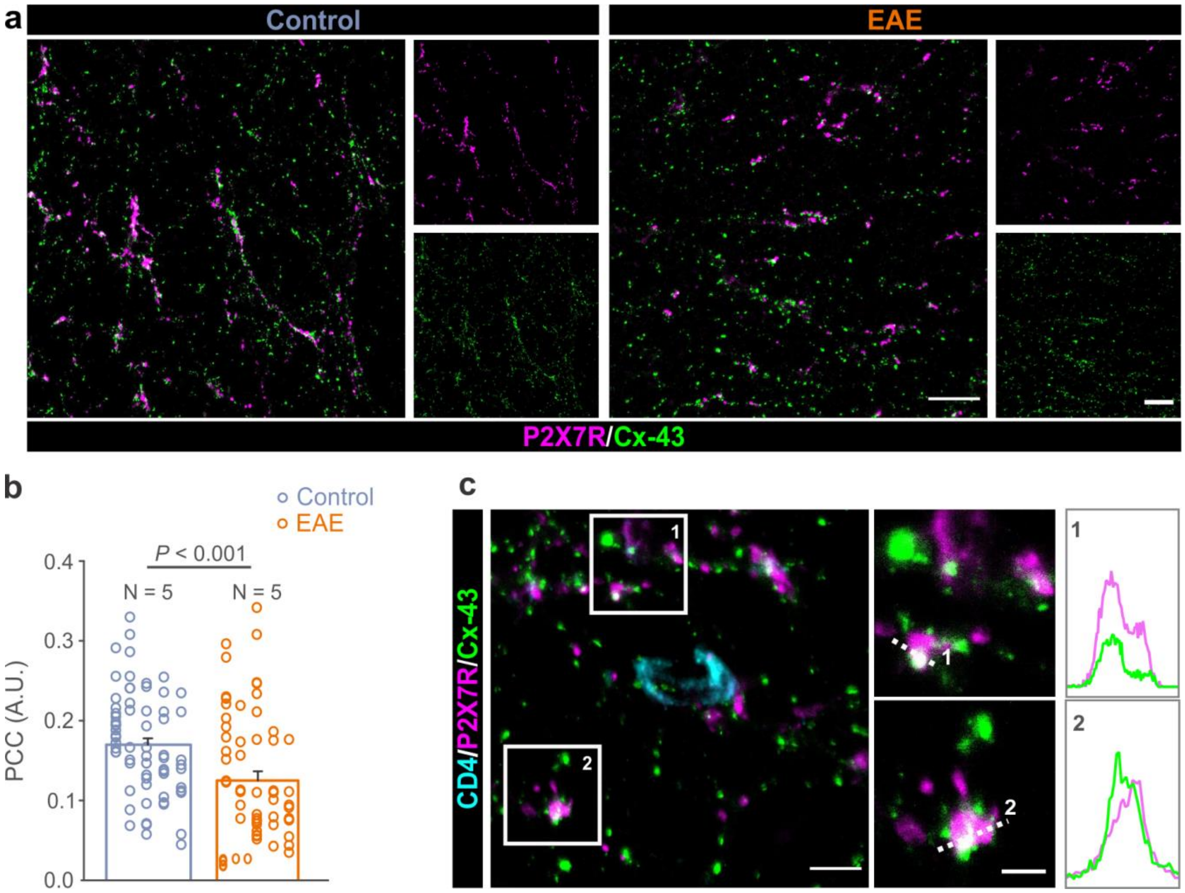

Supplementary Figure S4. P2X7 receptor colocalization with connexin-43 in the spinal cord white matter in EAE. (a) Representative confocal images of the spinal cord white matter immunostained for P2X7R (magenta) and connexin-43 (Cx-43, green) in control and EAE. Scale bars 20 µm. **(b)** Graph showing Pearson correlation coefficient (PCC) between Cx-43 and P2X7R in the white matter in control and EAE (Mann-Whitney Rank Sum Test, $P < 0.001$). N indicates number of animals. Each vertical dot plot corresponds to the data points obtained from the individual animal. Data are presented as mean ±SEM. **(c)** Confocal images of Cx-43 (green) and P2X7R (magenta) fluorescent signals in the close vicinity of infiltrated $CD4^+$ T cells (cyan) in the white matter of the spinal cord of EAE rats. Scale bar 5 µm. Numbered (1, 2) rectangles correspond to the regions presented on the right side. Scale bar 2 µm. Profile intensity plots of Cx-43 and P2X7R fluorescent signals are measured along each white dotted line.

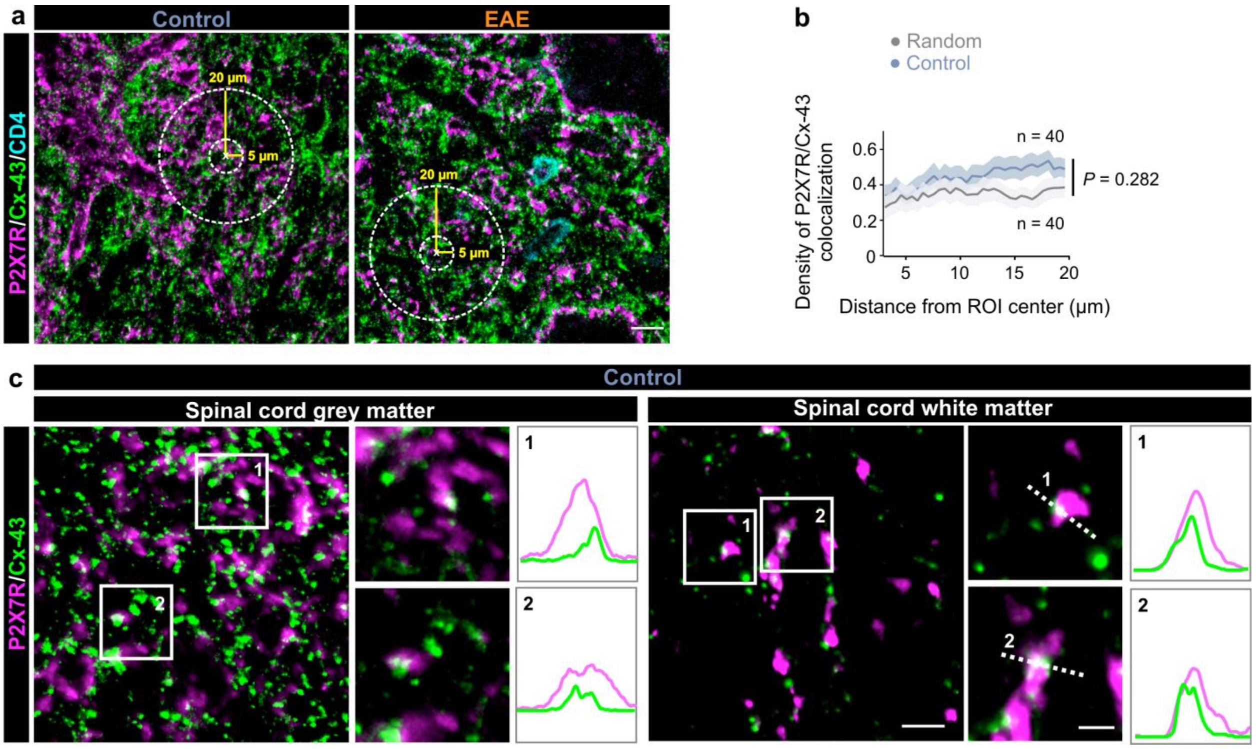

Supplementary Figure S5. P2X7 receptor colocalization with connexin-43 in the spinal cord of control healthy animal. (a) Confocal images of P2X7R (magenta), Cx43 (green) and $CD4^+$ T cell (cyan) immunofluorescent labeling in Control and EAE. Depicted regions of interests (ROI) are used for analysis of P2X7R, Cx-43 signal intensity and colocalization in the random region of interest (ROIs). Yellow and dashed white lines mark 5 µm and 20 µm radial distances measured from the center of ROIs. Scale bar 10 µm. (**b**) Graph showing density of P2X7R/Cx-43 colocalization in Control (pale blue) and random (grey) ROIs (two-way ANOVA, P = 0.282). Data are presented as mean ± SEM; n is the number of analyzed ROIs. (**c**) Confocal images of Cx-43 and P2X7R fluorescent signals in the white and the grey matter of the spinal cord of Control rats. Scale bar 5 µm. Numbered (1,2) white rectangles correspond to the regions presented on the right side. Scale bars 2 µm. Profile intensity plots of Cx-43 and P2X7R fluorescent signals are measured along each white dotted line.
